# Pose estimates from online videos show that side-by-side walkers synchronize movement under naturalistic conditions

**DOI:** 10.1371/journal.pone.0217861

**Published:** 2019-06-06

**Authors:** Claire Chambers, Gaiqing Kong, Kunlin Wei, Konrad Kording

**Affiliations:** 1 Department of Bioengineering and Department of Neuroscience, University of Pennsylvania, Philadelphia, Pennsylvania, United States of America; 2 School of Psychological and Cognitive Sciences, Peking University, Beijing, China; 3 Beijing Key Laboratory of Behavior and Mental Health, Peking University, Beijing, China; University of Muenster, GERMANY

## Abstract

Marker-less video-based pose estimation promises to allow us to do movement science on existing video databases. We revisited the old question of how people synchronize their walking using real world data. We thus applied pose estimation to 348 video segments extracted from YouTube videos of people walking in cities. As in previous, more constrained, research, we find a tendency for pairs of people to walk in phase or in anti-phase with each other. Large video databases, along with pose-estimation algorithms, promise answers to many movement questions without experimentally acquiring new data.

## Introduction

To successfully navigate any environment, a walker must adapt to the surface they walk on, avoid obstacles, change speed, and plan movements according to their goals [1,2]. Processes that impact walking behavior range from peripheral processes to high-level decision-making processes [3,4]. Walking, like all movement, often takes place in the context of other people. Humans, when walking, must often generate their actions according to the movements of people around them.

Coordination of movements with others during everyday activities helps to achieve shared goals efficiently and fluently, for example when playing sports, moving an object together, or walking side-by-side. In many cases, synchronization is needed to successfully carry out the task at hand. However, evidence from laboratory experiments suggests that synchronization between people does not only take place when it is required by the task. There is evidence for uninstructed movement coordination from laboratory experiments where subjects are asked to perform various artificial tasks, for example, sway pendulums, sit in rocking chairs, or spontaneously move their arms [5–11]. Coordination across individuals thus appears to be fairly ubiquitous, occurring even when it is not required by the task and there is no instruction to do so.

Previous laboratory-based studies have investigated if people walking side-by-side synchronize their movements. Walker synchronization is common when people walk side-by-side, and has been found in over-ground walking studies [4,12–14] and in treadmill walking studies [15–20]. For example, Zivotofsky and Hausdorff (2007) found that when people were asked to walk side-by-side, they walked in anti-phase (left-right, right-left), with the left leg of one walker aligned with the right leg of the other walker, without instruction. van Ulzen et al. (2008) tracked subjects during side-by-side treadmill walking and found both in-phase (left-left, right-right) and anti-phase (left-right, right-left) walking with preferred synchronization modes. In sum, previous results provide evidence that pairs tend to walk in phase or in anti-phase.

However, as with many movement science paradigms, it is not known if walker synchronization occurs in real world settings, outside of the laboratory. This is especially relevant for the walking studies in the laboratory: researchers have found a substantial difference in biomechanics between walking on the treadmill and walking on level ground [21,22]. The synchronization of pairs of walkers observed on treadmill might not generalize to real life settings.

There have been previous examinations of walker synchronization during over ground walking in laboratory settings. Most laboratory studies used motion capture systems applied to the limbs to quantify walking parameters. Research relied on reviewer ratings to address walking over ground in one study [14]. In a second lab-based study of over ground walking, accelerometers were attached to the trunk, and although their findings suggest synchronization between walkers, it is not possible to conclude whether walking is in phase or in anti-phase [23]. The general lack of work using motion capture outside of the lab is understandable since motion capture technologies were either low in precision, like smartphone sensors, or low in portability, with previous experiments requiring that subjects wear markers or recording equipment [24,25]. This made motion capture a challenge for studying walker synchronization in real life. Thanks to recent work on pose estimation from 2D video, it is now possible to perform marker-less pose estimation with only 2D video data as input [26–28]. These algorithms take as input a 2D RGB image and output an estimate of human pose in image coordinates with reasonable accuracy. Marker-less pose estimation applied to videos provides the opportunity to ask questions about naturalistic behavior outside the laboratory.

Here we apply pose estimation to online videos to ask how people synchronize their movements when they walk side-by-side. We used a state-of-the-art pose-estimation algorithm, OpenPose, to extract human body pose from 2D videos [26]. OpenPose finds joint positions of people in videos and fits a 2D skeleton model to each person in the image. We built a simple search algorithm to find video segments containing pairs of people walking side-by-side in YouTube videos. From the resulting video segments, we extracted the pose of people in the video. In order to track person identity through videos, we used our own simple implementation of a tracking algorithm that minimized the distance between pose estimates across frames. We then examined pose estimates to ask if people walking together synchronized their movements, using vertical ankle displacement to quantify walking angle and thus phase.

## Methods

### Video data

We wanted to obtain videos of people walking in a naturalistic setting. To find this data, we searched for videos on youtube.com using the search term “walking in” followed by the names of major cities: Bangkok, London, Paris, Dubai, New York, Singapore, Kuala Lumpur, Istanbul, Tokyo, Seoul, Hong Kong, Barcelona, Amsterdam, Milan, Taipei, Rome, Osaka, Vienna, Shanghai, and Prague. Videos were ranked by relevance by YouTube. We examined the most relevant videos for each search term that were over 20 minutes long. In our analysis, we included videos that contained pairs of people walking with low camera movement and where limbs of people in the video were uncropped. We selected videos that met these criteria from the 20 most relevant results on YouTube. Our methodology complies with the terms of service and use of YouTube. This study was approved by the University of Pennsylvania Institutional Review Board (protocol 827723).

The URLs from the 348 video segments included in the final analysis are included as Supporting information (S1 File). The files for YouTube videos with Creative Commons licenses and walking data for all videos can be accessed at https://figshare.com/articles/PONE-D-18-34216_Walker_synchronization/7722278. The data in the linked repository constitutes our minimal data set, which along with the information given in this manuscript is sufficient to replicate our analysis.

**Fig 1:**
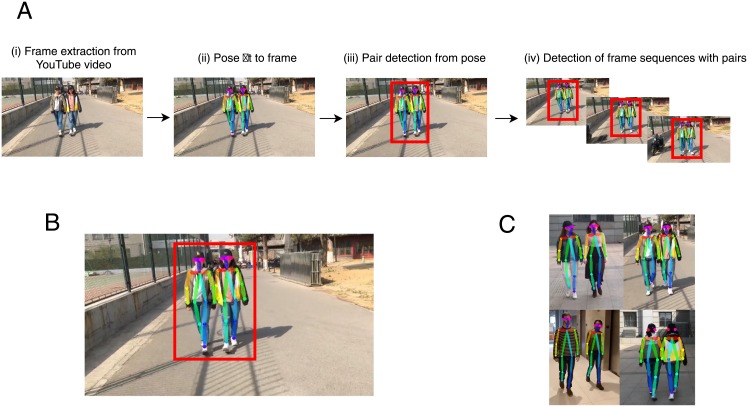
Search algorithm and algorithm outputs. (**A**) Flowchart describing stages of search algorithm used to find segments of videos containing pairs of walkers. We (i) extracted frames from subsampled videos, (ii) fit pose to the frames using OpenPose, (iii) detected pairs from pose estimates, then (iv) identified sequences of frames that contained pairs of people, resulting in a set of pose estimates from frame sequences. (**B**) Example video frame containing a walking pair with overlaid pose estimates. A bounding box highlights the persons identified as a pair. (**C**) Examples of walking pairs with overlaid pose estimates.

We provide examples of OpenPose outputs overlaid on video frames in Fig 1. The individuals pictured in Fig 1 have provided written informed consent as outlined in the official PLOS consent form to publish their image in the manuscript.

### Pose estimation

We needed a method to quantify the synchronization of walker pairs from 2D videos. We therefore began by extracting human pose from video frames, using OpenPose, a pose-estimation algorithm that detects body part positions from 2D RGB images [26]. OpenPose detects the position of the neck, shoulders, elbows, wrists, hips, knees, and ankles, as well as key facial points of eyes, ears, and nose. OpenPose assigns a unique person identity to each skeleton present in the image. In our analysis pipeline, we extract the pose of humans from individual frames of videos.

### Search algorithm

We first needed to identify video segments of people walking side-by-side from the downloaded YouTube videos. We therefore built an algorithm to search through YouTube videos and extract the particular segments containing pairs of people walking (Fig 1). First, we subsampled YouTube videos at a rate of 2 frames/second, then extracted pose estimates from video frames. Based on pose estimates present in a frame we detected pairs of walkers. Finally, we defined continuous sequences of frames that contained pairs of walkers for inclusion in our analysis.

From each frame of the subsampled YouTube video, we extracted human pose, resulting in a set of x, y coordinates indicating body part position and a person index. We included skeletons that contained at least 14 out of the total 18 parts. As movement synchronization is typically quantified by relative phase between two oscillatory motions, we created a measure of walking phase by using the ankle position. Therefore, we required that both ankles be detected for a given skeleton. This resulted in a set of pose estimates for each video frame with people present.

Using pose estimates from each frame, we then detected pairs of walkers on a frame-by-frame basis. We built a bounding box around each skeleton pair found in the image. To ensure that walkers were side-by-side, we selected pairs where the aspect ratio (height:width) of the bounding box was between 1:1 and 3:1, and where ankles of the pair were within 15% of the bounding box from each other in y-coordinates. To avoid data with low spatial resolution, we selected pairs where the height of the bounding box was over 120 pixels. To ensure that we analyzed pairs of adult walkers and not adult-child pairs, we only included pairs where the ratio between the larger and smaller skeleton was less than 1.25:1. This resulted in a set of detected person pairs from each video frame.

To produce video segments of pairs of walkers, we identified sequences of video frames that contained at least one pair of walkers for the whole duration of the segment, with a minimum segment lasting 2 seconds. We excluded pairs where two or more walker pair detections contained a common element (person) for 3 consecutive subsampled frames or more (1.5 seconds), which would suggest a group of walkers. We then extracted video segments of pair of walkers from the original YouTube video sampled at the original frame rate. We manually selected videos that contained non-occluded pairs of adults walking continuously toward or away from the camera on flat surfaces in well-lit conditions, where people were not carrying large objects or using an assistive device. In order to increase the quality of our data, we modified the duration of segments, either removing segments without walking pairs or increasing the duration of the segment until the point in the video where the walking pair was no longer visible. This resulted in video segments containing pairs of people walking together.

From each video segment, we needed to extract pose and track individuals through the video, in order to obtain cyclic walking signals for each person. We extracted pose estimates for all frames. Using the pose estimates, we tracked pairs of walkers through the frames of the video. We tracked pairs of walkers by first running the previous walker pair detection step on the first frame of the segment. We then minimized the distance between skeletons in the video from one frame to the next averaged across the key points present in both frames, $D=\sum_{i}^{n}\frac{1}{n}\sqrt{{\left(x_{i,j}-x_{i,j+1}\right)}^{2}+{\left(y_{i,j}-y_{i,j+1}\right)}^{2}}$ where *i* is a keypoint index, *j* is a frame, *n* is the number of keypoints that are present in both frames, *x* is the x-coordinate of a key point and *y* is the y-coordinate of the key point. To track a given person, we iterated through the frames of the video and minimized the distance for each frame pair. Common problems were switches in person identity across frames and failure to accurately track the ankle. We therefore manually selected videos for inclusion in our analysis where the algorithm successfully tracked people through frames of the video.

### Data analysis

To quantify walker synchronization from pose estimates, we analyzed the vertical displacement between the ankle joints, using this as a way of quantifying the relative walking phase. Our analysis was adapted to measuring walking phase from people walking toward or away from the camera. We chose to analyze the video data in this way firstly because inspection of the video data revealed that walkers more often moved toward or away from the camera rather than across the frame. Second, visual occlusion between walkers prevented us from accurately extracting the pose for both walkers when they walked side-by-side in profile, with one walker occluding the other in the video frame. Although the profile view provides greater variance in leg angle and in this sense is less prone to noise than metrics extracted from the frontal/dorsal view, occlusion would prevent us from finding and then analyzing videos with a profile perspective. Therefore, analyzing vertical ankle displacement extracted from the frontal/dorsal angle was a strategy which was adapted to most of the data available in our video data set and also allowed us to avoid problems related to occlusion.

In order to extract walking phase from pose estimates, we processed the data as follows. We first replaced missing values using linear interpolation. We did not have control over video recordings and so, in most video segments, the distance between the camera and the walkers was not fixed. We therefore needed to detrend the ankle coordinates. To do so, within each frame, we computed the distance of the ankle relative to a reference body part and divided this distance by the height of the skeleton. We selected the neck as the reference part since this is typically the least occluded part. We combined this data across frames to form a time series of detrended ankle coordinates. We handled outliers by replacing points which were two standard deviations or more from the mean with linearly interpolated values. We then normalized the signal by subtracting its mean and dividing by its standard deviation. We low-pass filtered the ankle displacement signal using a third order Butterworth filter with a cutoff frequency of 2 Hz. This resulted in a signal from which we could extract the vertical ankle displacement and walking phase.

**Fig 2:**
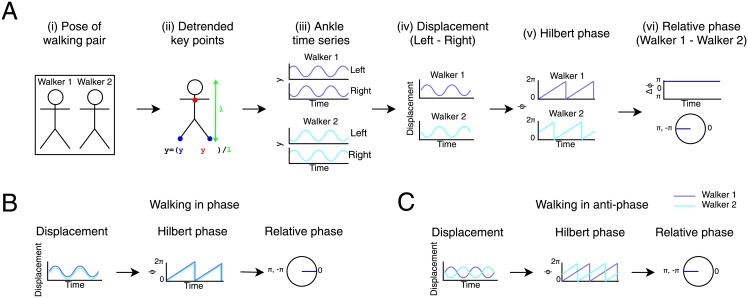
Analysis of relative phase illustrated. (**A**) Main stages of data analysis performed on pose estimates from videos of walking pairs. (**i**) We tracked the pose of Walker 1 and Walker 2 from each video clip. For details, see Fig 1A. (**ii**) We detrended key point coordinates for each walker within frames by subtracting a reference point on the same skeleton (y_ref_, the neck) from the ankle coordinates (y_a_) and dividing by the length of the skeleton (l). (**iii**) This resulted in a time-series of y-coordinates for the left and right ankles for Walker 1 and Walker 2. (**iv**) For each walker, we subtracted right from left ankle coordinates to give a time series of the left-right displacement between ankles. (**v**) We used the Hilbert phase of the displacement signal to approximate walking angle for each walker. (**vi**) Finally, we computed the difference between the phase of Walker 1 and Walker 2 to obtain a time series of the relative phase of the walkers (upper panel) from which we computed the distribution of relative phase on the circular axis (lower panel). For a similar analysis, see [17] (**B**) Pair walking in phase. The ankle displacement signal overlaps for Walker 1 and Walker 2, as the pair walks perfectly in phase. There is therefore a constant relative phase of 0. (**C**) Pair walking in anti-phase. The displacement signals are in antiphase with each other. There is a constant absolute phase difference of *π* between the signals in the Hilbert phase time series, leading to a relative phase distribution with a peak at *π*.

For each pair of walkers, we wanted to analyze the walking phase relationship using the positions of the ankles. Our analysis of walking phase is described in Fig 2 and resembles that of [17], which was originally applied to walking angle measured from optical tracking data. We computed the relative ankle displacement by subtracting the right ankle position from the left ankle position. We then computed the Hilbert phase of the displacement signal. The phase signal, which we use as a proxy for oscillatory walking motion for one person, varied between −*π* and +*π*. The relative phase between walkers was simply computed by subtracting the walking phase of one walker from the other. We also wrapped the phase difference so that it could not extend outside −*π* and +*π*. Thus, the relative phase was a continuous circular variable that wrapped at the +*π*. This resulted in a time series of relative phases for each pair of walkers. We analyzed the distribution of relative phase for each pair of walkers by computing the mean phase angle computed using a circular mean statistic.

In order to combine estimates of walker synchronization across different videos, we used circular statistics to compute mean of the relative phase distribution and the variance of the relative phase distribution from each video segment. Using the R package movMF, we then fitted a mixture of von Mises distributions to the distribution of mean relative phase extracted from all videos, and identified the peaks of the best fitting distribution for up to four components. The mixture of von Mises distributions is a circular approximation of a mixture of Gaussian distributions. The aim of this analysis was to quantify the location of the peaks of the relative phase distribution. Peak phase of 0 would mean that walking in phase is a dominant mode of walker synchronization, and peak phase of +*π* would mean that walking in anti-phase is a dominant mode.

We extracted the frequency of walking by examining displacement between left and right ankles in y coordinates. The displacement signal is positive when the left ankle is above the right and negative when the left ankle is below the right. This displacement signal is cyclic, and one cycle represents two steps. We extracted the frequency by computing the peak value of the discrete fast Fourier transform. We doubled this frequency in cycles/second to obtain the walking frequency conventionally defined as steps/second.

In our video data, we had no control over how the video was recorded. For example, the camera was often in movement and camera angle was variable, both of which could have an influence on our measure of ankle position. We therefore wanted to ensure that pose estimation did not produce results on frequency and relative walking phase were unbiased.

We verified that the mean relative phase extracted from pose estimates was consistent with the mean relative phase extracted from labels of foot strike timing labels provided by two human labelers. Our “ground-truth” data set consisted of 5-second-long segments from 43 randomly-selected videos from our final data set. We built a signal that accurately reflected the walking angle using human labels of foot strike timings. We then compared the relative phase computed from ‘ground-truth’ human labels with the relative phase computed from pose estimates. We performed the same analysis on a signal constructed from human labels that was applied to the ankle displacement signal computed from pose estimates. Agreement between the analyses applied to human labels and pose estimates will suggest that pose estimates allow unbiased estimation of relative walking phase.

We required a signal that would approximate walking angle which we could construct from human labels. We therefore labelled videos with foot-strike timing events for the left and right feet of each walker. We annotated videos using a video annotation tool (VideoAnt, https://ant.umn.edu/) by manually labelling the timing of foot strikes for each walker (for example, the left foot of Walker 1 struck the ground at 00:02, the right foot of Walker 1 struck the ground at 00:03). This resulted in a set of left and right foot strike events for the two walkers in each video clip. Note that the foot strike is one of the most salient kinematic event for locomotion, and its timing is easy to estimate [29,30].

From these timing events, we generated a sinewave representing the walking angle for each walker. When we walk, the walking angle is at its maximum when either foot strikes the ground. To construct a signal that would behave in this way, we built a piecemeal sinewave that oscillated between -1 and 1: -1 for each left foot-strike timing event and 1 for each right foot-strike timing event. We analyzed the resulting signal as we did the y-ankle displacement signal from the pose estimates. We computed the *R*^*2*^ to assess agreement between the analysis of frequency and mean relative phase from pose estimates and from human labels.

We wanted to ensure that the ages of pairs found in videos were not biased to a certain age range, e.g., youth. For the videos in our final data set, we estimated the ages of walkers in each pair to the nearest decade.

We wanted to examine the effect of tactile feedback on synchronization. Therefore, we manually labelled videos according to whether there was hand contact between walkers or no hand contact between walkers.

## Results

We asked if people synchronize their walking when they walk side-by-side in naturalistic settings. To do so, we analyzed videos found on YouTube. Within YouTube videos, we searched for video segments with pairs of people walking. From video segments, we extracted the pose of each member of the walking pair. In order to examine walker synchronization, we analyzed displacement between left and right ankles for each member of the pair. Based on the displacement signal, we extracted the walking frequency and mean relative phase for each pair to examine walker synchronization.

To find relevant videos, we searched for videos on youtube.com using the search term ’walking in’ followed by the names of major cities. Our search resulted in 363 videos. We excluded 113 videos because they did not include footage of pairs of people walking. We excluded 48 videos because of large amounts of camera movement, cropped video frames, camera angles which prevented pose estimation, and poor visibility. We excluded 3 videos because the format of the video was not suitable (a large amount of occluding text on the screen and panoramic videos). After screening, we included a total of 199 videos, which gave us a reasonably sized dataset for quantifying synchronization.

**Fig 3:**
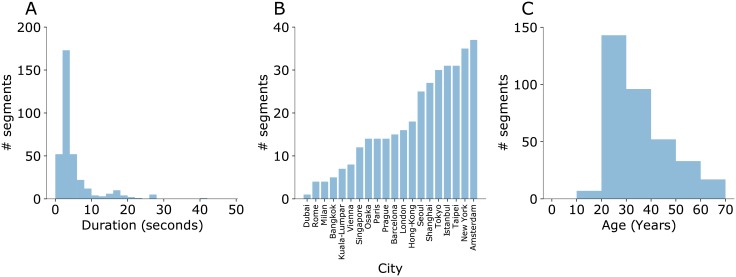
Descriptive analysis of videos. (**A**) Distribution of duration of video segments in seconds. (**B**) Number of video segments for each city we searched for. (**C**) Distribution of estimated age of walkers averaged across the pair in years.

Within these videos, our algorithm identified 888 video segments with pairs of people walking lasting at least 2.5 seconds. Some segments were unsuitable because 1.) the segment did not contain pairs of adults walking continuously towards or away from the camera in well-lit conditions (258 segments), 2.) the segment included large amounts of camera movement, occlusion, or cropping of body parts from the video which prevented successful pose-tracking (197 segments). In the end, we extracted pose from the 433 suitable video segments containing 441 pairs of walkers, among which pose estimation or tracking failed for 93 walking pairs. Thus, our final sample for walking analyses include 348 video segments with a mean duration of 4.73 seconds (SD = 5.99, Fig 3). Walkers in the video had a mean age rating of 30.66 years (SD = 12.34). Although most walkers were in the young age range of 20~40 years (71%), we still had 29% of walkers in the range of 40 to 70 years. Thus, we have a reasonably representative dataset to investigate our questions.

**Fig 4:**
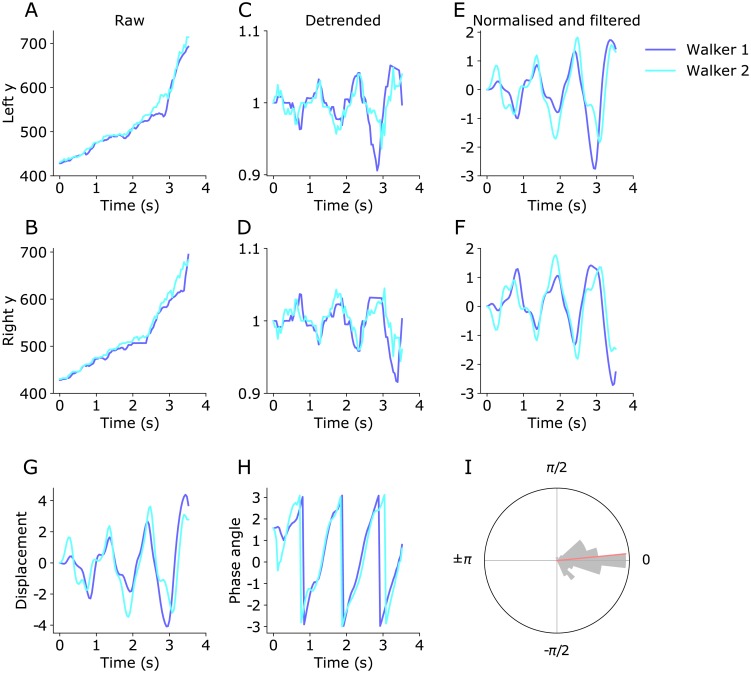
Extracting relative phase from the image y-coordinates of the left and right ankles. Walker 1 and Walker 2 from the same pair are presented on the same panel (video #13, see S1 File). The strong overlap between the signals shows that the pair is walking in phase. (**A, B**) Raw y-coordinates of the left ankles (A) and right ankles (B) with Walker 1 and Walker 2 shown in different colors. (**C, D**) We then detrended the signal by subtracting the y-coordinate of a reference body part, the neck, and dividing by the height of the walker. This gave a signal that was invariant to the position of the walkers in the frame and their size. (**E, F**) We removed outliers, normalized and low-pass filtered the signal. (**G**) Using the normalized y-coordinate signal, we subtracted right ankle coordinate from the left ankle coordinate to give a displacement signal for each walker. (**H**) We computed the phase angle by taking the Hilbert transform of the displacement. (**I**) We examined the distribution of the relative phase between walkers by computing the mean of the distribution (red).

We wanted to quantify the degree of synchronization in the walking phase of each pair. To do so, we examined the phase relationship between two oscillatory walking patterns, using the signed y-displacement between left and right ankles. From the time series of left and right ankle positions in y-coordinates (Fig 4A and 4B), we extracted the cyclic motion of walking for each member of a walking pair. We first detrended the y-coordinates of the left and right ankles (Fig 4C nad 4D). Then we normalized and low pass filtered the detrended signal (Fig 4E and 4F). For each walker, we computed the displacement between the left and right ankles (Fig 4G), then computed the Hilbert phase of the displacement time series (Fig 4H). Finally, we computed the relative phase between Walker 1 and Walker 2. We examined the distribution of the relative phase to quantify walker synchronization (Fig 4I). The representative video segment in the Fig 4 showed a predominant in-phase synchronization as the relative phase clustered around zero. The ability to estimate relative phase from videos enables us to estimate synchronization behavior in the population.

**Fig 5:**
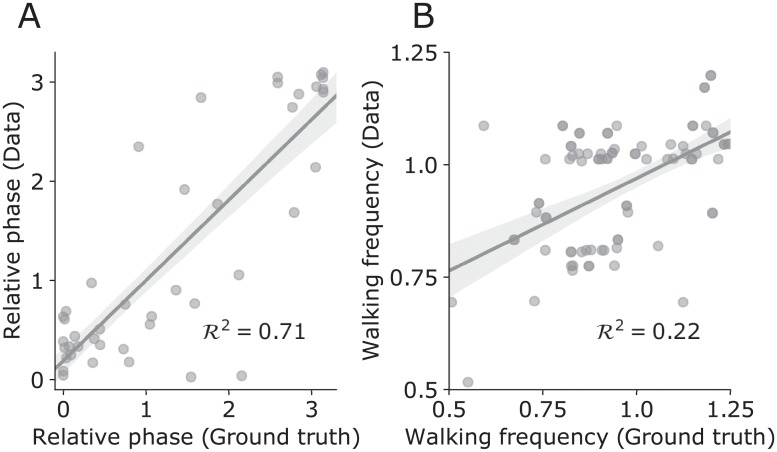
Comparison of relative phase and walking frequency computed from pose estimates and signals extracted from human labels of foot strike timings from 43 5-second long videos. (**A**) Mean relative phase from pose estimates as a function of mean relative phase from human-labelled data with overlaid linear fit (shaded area = 95% confidence interval). (**B**) Walker frequency from pose estimates as a function of walker frequency from human-labelled data with overlaid linear fit (shaded area = 95% confidence interval).

We wanted to validate the outputs from pose estimates. We thus compared the walking frequency and mean relative phase computed from pose estimates with those extracted from a ground truth signal. We found reasonable agreement between the ground-truth estimates and pose estimates (Fig 5, relative phase: R^2^ = .71 (N = 43); walker frequency: R^2^ = .23 (N = 43)). Therefore, despite the variability of recording conditions, we were able to meaningfully extract information about walking patterns from pose estimates.

As an additional validity test of our method, we tested if its walking frequency estimate agrees with the typical walking cadence in the literature. We thus measured walking frequency from ankle displacement signals (Fig 5C). The average walker frequency was 1.85 Hz (SD = .32), which is very similar to the average of ~2 Hz previously reported in the literature [31]. The range of walking frequency was approximately from 1 to 2.5 Hz, appearing to reflect the cadence in naturalistic settings. In fact, the frequency within pairs was almost identical for some walking pairs, shown by points close to the diagonal line in Fig 5D. The frequency of walkers differed by less than.1 Hz in 66% of walking pairs. This was not the case for all walking pairs, though this is understandable as the walking pairs differed (e.g., heights) and our pose estimation is inevitably plagued by noise.

**Fig 6:**
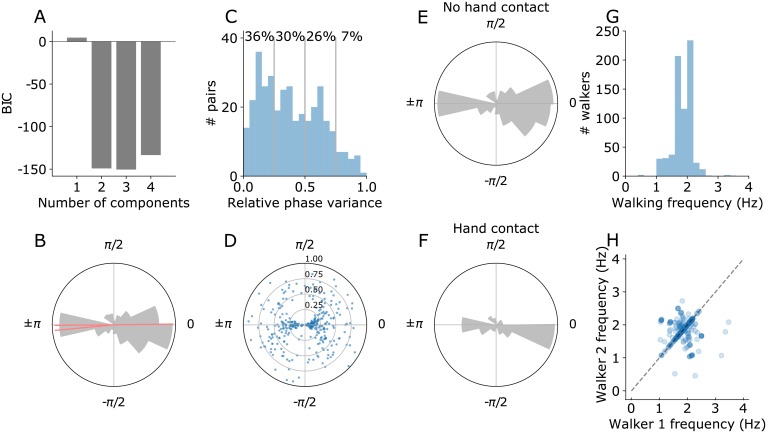
Group results on relative phase and walking frequency. (**A**) We fitted mixture of von Mises distribution models to the distribution of mean relative phase, varying the number of peaks of the distribution. The best-fitting model contains three peaks. (**B**) The distribution of the mean relative phase across pairs of walkers and means of the best-fitting mixture of von Mises distributions (red). (**C**) Histogram of the variance of the relative phase signal extracted for each walking pair. Vertical lines show relative phase variance of.25,.5, and.75, with the proportion of the data that was in these intervals (**D**) Scatter plot of the relative phase variance on the radial axis as a function of the mean relative phase for each pair. Pairs are strongly synchronized with mean relative phase near 0 or ±*π* radians when variance is low. (**E**) The distribution of the mean relative phase across pairs of walkers where there was no hand contact (N = 288). (**F**) The distribution of the mean relative phase across pairs of walkers where there was hand contact (N = 60). (**G**) Distribution of walking frequency, quantified using ankle displacement (Mean = 1.85 Hz, SD = .32). (**H**) Scatterplot of walking frequencies of walkers within each pair.

We asked what the overall synchronization is in our sample as a whole. For an analysis that combined data across video segments, we quantified the mean relative walking phase for each video segment and examined its distribution across all segments. To quantify the most prevalent relative phases, we fit a mixture of von Mises distributions model to the distribution of mean relative phase, comparing models with one to four peaks. We found that the model with three peaks provided the best fit to the data, as quantified by Bayesian Information Criterion (Fig 6A). The means of the three components or peaks of the model were.01, -3.05, and -3.13 in radians with concentrations (normalized κ) of.17,.80, and.02 respectively (Fig 6B). The component with approximately zero mean shows that pairs walked in phase with each other. The other peak close to *π* show that pairs also walk in near anti-phase with each other. Note, the two peaks near *π* are close to each other and, accordingly, the two-peak model is comparable to the three-peak model in BIC. We have thus shown that people walking in pairs walk in phase or in anti-phase with each other.

A second indicator of synchronization is a stable relative phase signal between walkers. Low variance between walkers indicates phase locking in walking patterns. Phase locking can occur at different mean phases. High variance indicates a lack of phase locking that is consistent throughout the segment, but here is difficult to separate from the effects of noise on pose estimates. We analyzed the distribution of the relative phase variance computed for each walking pair (Fig 6C). First, we observe that the distribution is positively skewed with more walking pairs exhibiting low variance. Therefore, there is evidence of phase locking for many walking pairs in our sample. Next, we examined the variance of relative phase as a function of the mean relative phase for each walker (Fig 6D). Walking pairs with lower variance (36% of walking pairs, relative phase variance < .25) phase locked close to 0 or ±*π*. The spread of the mean relative phase increases with the variance of the relative phase, as phase locking between walkers becomes less pronounced. Therefore, we find phase locking indicated by walking pairs with low variance of relative phase. In most pairs with low variance of relative phase (< .25), phase locking occurs close to 0 and ±*π*. Therefore, our analysis of the relative phase variance indicates phase locking in near phase or near anti-phase in 35–70% of walking pairs in our sample.

Walkers synchronize their movements using sensory feedback. Therefore, we asked if tactile feedback is crucial for synchronization to occur, by separately analyzing videos with hand contact between walkers (N = 60) and without hand contact between walkers (N = 288). We observe synchronization indicated by peaks at 0 and ±*π* both in cases with and without hand contact (Fig 6E and 6F). Therefore, tactile feedback is not necessary for synchronization to occur.

## Discussion

We asked how people synchronize their movements when they walk side-by-side, by analyzing pose estimates extracted from online videos. We searched for videos on youtube.com using the search term ’walking in’ followed by the names of major cities. We analyzed the relative phase and walking frequency computed from the vertical displacement between the ankles in each video. We validated our analysis from pose estimates through comparison with ground-truth data and found reasonable agreement between estimates of mean relative phase and walking frequency. The mean frequency of walkers was 1.85 Hz, close to the average of 2 Hz reported in the literature [31]. We found that the distribution of relative phase across video segments contained prominent peaks near 0 and *π*, which reflects a tendency for pairs of walkers to walk in phase or in anti-phase with each other. Using pose estimation applied to online videos, we confirmed findings from more constrained laboratory experiments on a larger sample and in more naturalistic conditions. We have thus shown that a pose estimation algorithm applied to 2D videos can be used to address questions about naturalistic movement.

The trend for walking in-phase or in anti-phase in our data is consistent with previous laboratory studies from treadmill studies [17,18,20]. We found that the predominant mean phase difference across walkers is at 0 and ±*π* radians, suggesting that in-phase and anti-phase walking occur for different walking pairs. In agreement with our findings, when pairs walked on treadmills, phase locking occurred close to 0 and ±*π* radians [17,18,20]. However, shifts away from in-phase and anti-phase locking were also found for some pairs [17]. This does not reflect the overall trend in our data set, but it is possible that some pairs with higher variance of relative phase show this shifted pattern of phase locking intermittently. Our examination of the variance of relative phase suggests that not all walking pairs synchronize, with some pairs having high relative phase variance. While this cannot be separated from the effect of noise in pose estimation data, this is also consistent with findings from laboratory-based studies where it was found that 50–60% of pairs synchronized. Our findings are, therefore, globally consistent with results on walking on treadmills.

While findings from treadmill studies provide evidence for walker synchronization, they do not provide direct evidence for walker synchronization in naturalistic unconstrained situations. Treadmills are set at a fixed walking speed [17] and biomechanical studies have found that normal ground walking is different from treadmill walking [21,22]. Therefore, manipulation of walking speed and the very fact of walking on the treadmill might impact the synchronization behavior and prevent generalization of findings to more natural situations.

Our study builds on previous work on walker synchronization during over ground walking. In previous work on over-ground walking, human raters judged pairs as exhibiting synchronized walking on an ordered scale, providing qualitative evidence for in-phase and anti-phase synchronization. A second study quantified walker synchronization during over-ground walking using accelerometers applied to the trunk [23]. Synchronization was found but could not be identified as in-phase or in anti-phase. Our method allowed us to address if pairs walked in phase or in anti-phase during unconstrained, naturalistic walking, using video analysis. In these videos, people walk with their “preferred” speed, and their leg lengths presumably vary widely. Despite the lack of experimental control, we still find evident phase entrainment between walking patterns with findings of both walking in phase and in anti-phase. Hence, our study provides solid evidence for synchronization in walking pairs. Globally, the trend of walker synchronization both in phase and in anti-phase is consistent with previous findings of walker synchronization, with our results supporting the notion that walker synchronization is common in real-world settings.

We developed a pipeline to study walking behavior from videos. Using this pipeline, we were able to extract sufficient information to carry out a group analysis across hundreds of video segments. Outside the scope of this work, various interesting questions arise on how synchronization occurs in the real world. One may ask how sensory feedback, biomechanics, and social factors influence walker synchronization. Addressing most of these questions in our data set would require a more detailed analysis of individual data, which is precluded by the level of noise in our data and the lack of longer video segments in our data set. Larger data sets and more precise pose estimation algorithms will make many of these investigations possible in future.

Previous work has examined the necessary conditions for walker synchronization. Sensory feedback is needed for synchronization behavior to occur. In order to synchronize movement, pairs of walkers use auditory, visual, and tactile sensory feedback from their partner [12,14,23]. Some laboratory results suggest that tactile feedback is more effective in producing synchrony during over ground walking than visual or auditory feedback [14,23] and there is evidence that walkers can use tactile force information alone to coordinate their movements during treadmill walking [12]. During walker synchronization, authors have observed regularities in tactile interaction forces [12]. Here, we find that synchronization occurs in cases with and without hand contact, so while hand contact is sufficient for synchronization to occur, it is not necessary under real-world conditions. Therefore, pose estimation applied to online videos may provide information on the role played by sensory feedback. However, in this case this methodology serves as a complement rather than a substitute for laboratory experiments, since our methodology does not allow us to experimentally control sensory feedback for examination of the causal determinants of walker synchronization.

In the laboratory, biomechanical and environmental parameters modulate synchronization behavior, such as differences in leg length, walkway slope and over-ground speed between partners [16,18]. In future studies, pose estimation may be used to understand in greater detail the factors that influence walker synchronization under real-world conditions. With larger data sets and improved pose estimation, we may be able to address the influence of biomechanical parameters that can be measured directly from pose estimates and environmental parameters measured from videos, such as the influence of leg length or walkway slope on synchronization behavior.

Questions also arise on the interpersonal factors that influence synchronization. Synchronization between pairs who intentionally walk together is a specific case, where walkers share the goal of walking side by side. This presumably captures much of the data analyzed in this work. However, synchronization could occur across a group of walkers of more than two people, as there is evidence of synchronization in collective animal behavior [32,33]. Synchronization could also generalize to cases where people are walking in proximity but not intentionally walking together as a pair or group. This is suggested by walker synchronization in laboratory settings occurring spontaneously in 50–60% of healthy individuals [14,17,18]. Future work could use pose estimation to assess the generality of synchronization across walkers in real world settings.

Although pose estimation is a promising tool for movement science and related applications, its application at present involves limitations. One challenge is the presence of different sources of variability in videos. Due to imperfect pose estimation, our measure of ankle joint position varied around the true position. To remove noise, more sophisticated forms of denoising than the standard signal processing techniques used here can be applied. For example, measuring the contribution of different noise sources would allow the application of deconvolutional techniques to separate the walking phase signal from noise. Another problem is that camera angle, which constantly changes in the videos that we used, influenced the ankle displacement variable. Signal cleaning that uses machine-learning based predictions from, for example, recurrent neural networks would allow the algorithm to learn the mapping of 2D-joint positions to walking phase [34]. Although standard techniques were sufficient for our purposes here, we anticipate the need to apply more sophisticated techniques to address questions that require more precise measurements when using real world data.

A second limitation is that most existing pose-estimation algorithms output two-dimensional pose estimates [26,27]. Without depth cameras, for now it is impossible to measure true three-dimensional kinematic variables. For example, in the present study, this prevented us from measuring walking phase directly from joint angles. Answering questions about naturalistic movement and developing usable applications generally requires estimation of a subjects’ three-dimensional pose. Existing algorithms that fit a three-dimensional body model to two-dimensional images may provide a solution [28,35]. Unlike commonly used forms of motion capture, such as Kinect and Vicon, these newer algorithms need only 2D images from a single camera as input to provide 3D pose estimates and therefore represent an advance on existing motion capture systems. However, they are, for the most part, inaccessible to those without expertise in the field of computer vision. The release of these algorithms in the form of more accessible software will contribute to the advancement of movement science and many fields in behavioral science.

A third challenge is continuous tracking of person identity in videos. Pose estimation algorithms provide joint position coordinates for each frame but do not track person identity throughout a video. Simple tracking algorithms that minimize distance across frames often fail because large amounts of occlusion present in natural scenes. Improvements of optimization methods for tracking algorithms and integrating pose estimation with tracking on whole videos will allow more successful analysis of movement in natural and dynamic scenes [36,37].

Besides algorithmic solutions, we can devise practical solutions using specialized setups to meet the above challenges. For example, we could carefully place multiple cameras in naturalistic settings. Alternatively, we could place lab-grade sensors on people in the real world. While all these approaches are feasible and have been used in past research, having scalable approaches that can leverage large datasets is particularly promising.

Many real-world applications are calling for automatic pose estimation based on videos. Improved pose tracking will allow the field to quantify movement variables from easily obtained data, thus paving the way for a host of video-based applications in fields including medicine and sport [38]. Movement tracking applications will allow us to better quantify the parameters of movement indicative of disease and thus will enable us to build objective, inexpensive, and affordable video-based diagnostic software that can replace expensive in-person evaluations. Similarly, pose tracking can be used to quantify the success of physical therapy interventions and will provide an objective standard that will help to guide therapists. Applications for sports, based on the parameterization of the best possible movement and other relevant variables related to fatigue or injury, include “virtual coaching” where an application can instruct users on when to train or how to best execute a movement from video recordings. We expect that such applications will soon be integrated into standard practice within these fields.

Movement science stands to gain the most from adopting pose estimation from 2D videos as part of its standard toolkit. Movement science and the field of psychology more generally have been limited by small data set sizes and a set of overly constrained laboratory tasks that may invoke qualitatively different mechanisms to those at play during real world tasks [39,40]. Examination of 3D kinematics of real-world movement data from 2D video will capture important aspects of behavior unseen during simple laboratory tasks. Such an examination alone may be sufficient to inform models. Pose estimation can be used in combination with an experimental approach to constrain the kinds of experiments we do in the laboratory, and to provide evidence for or against models based on real-world data. Using 2D videos will make large amounts of kinematic data easy to collect and will remove the need to collect new data in some cases, since existing 2D video data sets can be used. To progress, movement science must embrace a more ecological approach that emphasizes movement as it occurs “in the wild” [38,40] and we expect that pose estimation from video will be key to this process.

## Supporting information

S1 File Video information. CSV file containing: video # index, video URL, start of video segment in seconds, duration of video segment, name of corresponding file in database (pose estimates and video), video license information, and presence of hand contact.
(CSV)
